# Obtunding Dentin by Local Applications

**Published:** 1913-09-15

**Authors:** Elmore W. Elliot

**Affiliations:** Chicago


### OBTUNDING DENTIN BY LOCAL APPLICATIONS.

BY ELMORE W. ELLIOT, P.H.G., D.D.S., CHICAGO.

The dentist who is going to be the most successful in eliminating pain in his dental operations must of necessity be an electic. By this I mean he must use all of these various methods, depending on the case at hand. With some patients nitrous oxid and oxygen properly administered produce ideal results. In certain cavities, such as a small gingival, either warm or cold air will aid materially. In fact, in many such cases all pain is eliminated. I have found, however, that with certain patients more satisfactory results to all concerned are obtained by the topical application of remedies. In my own practice I find that some patients abhor the thought of taking gas, and cold air in large, deep-seated cavities in my hands has been disappointing to say the least. In these cases phenol compound gives great relief by applying the remedy directly to the cavity as often as it seems to be necessary.

It is my invariable custom to warm my remedies before applying to the cavity. This is done by passing the same through the flame of the bunsen burner. This will be greatly appreciated by the patient.

We have able authority which claims that normal dentin is without sensation. Whether this be correct or not, I am unable to say; but neverless I do know that the dentist has to do with conditions where the dentinal fibrillae have been subjected to continued irritation, with the result that the dentin is often hypersensitive.

We are greatly indebted to Dr. J. P. Buckley for the many valuable formulas which he has so freely given to us. One of these which has given me great satisfaction is the following: Zinc chlorid 20 grains; 4 drams each of alcohol and chloroform. This is not a panacea for all cases; but in large cavities, in molars and bicuspids, which approach the pulp, its use is very gratifying. In this formula the irritating property of the zinc chlorid is controlled by the chloroform, the alcohol being necessary to effect a solution.

Frequently cases present in the mouths of young children where the first permanent molar is badly decayed, with the pulp fortunately still alive. How to treat and protect this pulp is often a very difficult problem. If the cavity is such that a remedy can be sealed in, I frequently use the official oleate of cocain. This is rather a new preparation, having been introduced for the first time in the last edition of the United States Pharmacopeia. The cavity should be kept dry, preferably by the use of the rubber dam, when all dentine which you wish to obtund should be covered with the remedy. Dry cotton is then placed over it and the cavity sealed with a temporary cement to prevent pressure. In case the oleate of cocain cannot be obtained, I suggest the following formula which will act equally well: 2 grains each of the alkoloidal cocain and thymol, added to ½ dram sterilized liquid petroleum. This formula has the added advantage of being a disinfectant as well as an analgesic.

One advantage of obtunding sensitive dentin by the topical application of remedies is that most drugs used for this purpose, besides possessing the property of local analgesia, for which purpose they are here used, also possess the property of disinfection. We, therefore, sterilize the dentin at the same time we obtund the sensitivity of the dentinal fibrillae. We should ever bear in mind in all methods of obtunding hypersensitive dentin, the possibility of injuring the pulp. There is no question in my mind but that many pulps have subsequently given trouble, because of the lack of proper sterilization of the dentin before the insertion of the cement base and the final filling. The products of albuminous decomposition, if not removed, act as irritants to the pulp, inducing pathologic disturbances frequently of such a character as to demand the removal of the organ.

I believe dentists should endeavor to perform operations with as little pain as possible, yet I question seriously whether it is best with certain patients to eliminate all pain in every operation. Pain is our indicator, pain should be our guide. We should ever bear in mind in the use of drugs and remedies for the purpose of obtunding sensitive dentin that suggestion is as valuable here as it is in connection with other measures for accomplishing this end, and I repeat that there are few operations where the patient will appreciate our efforts more than in the careful, thoughtful and judicious use of any method for making our dental operations less painful.

### DISCUSSION

Dr. C. N. Johnson: In all these papers I believe one significant thing was mentioned, and that was the psychological effect of all of these procedures. It is the most important effect that has been produced, and by way of illustration I would like to refer to a certain incident that occurred in Chicago when cataphoresis was at its height. A gentleman had a tooth filled with absolutely ideal results. He got out of the chair and said: "I shall never have a tooth filled by any other method." He came in soon after for another operation. It happened that the cataphoric outfit was out of commission, but the dentist had a head on him. He seated the patient in a chair, made the application the same as before without any cataphoric action whatever, prepared the cavity and filled the tooth. When the patient got out of the chair, he said: "I declare it is better than I thought it was, and I've decided never to have a tooth filled any other way." That is an actual fact. A great deal of the analgesia of today is the same thing. A man who has that psychic power in him does not need to use an analgesic every time he puts his patient in the chair. I believe I can lay claim to as much sympathy for my patients as any man, and I deplore the necessity of giving pain. But if I should introduce into my office an outfit of that kind, I do not believe I would use it very much oftener than Dr. Kabell. I believe we can handle the patient without asking him to take analgesics in every case. I do not believe it is to the advantage of the patient to encourage the use of these things in the office. I believe we should do everything we can to allay the apprehension many individuals have of dental operations, and it is a splendid thing to show the patient that we are willing to do something to relieve them of discomfort. I have this conviction as firmly as anything, that if you and I will study human nature, and study kindness and delicacy of manipulation, showing the patient that we have an interest in him, the patient will co-operate with us, and we will to a large extent remove that dread of operations, and thus get along without all this paraphernalia. I do believe that in ten years from now we will hear less of analgesia than we do tonight. I do not believe it is for the benefit of the people to encourage them in these things. Somebody said that pain was beneficial. I do not believe in carrying it so far that it becomes a shock. If we have a capital operation by all means administer these things intelligently, and get the most out of them. My patients complain as much of other things as they do of sensitive dentin. They complain of the inconvenience as much as they complain of the pain. They complain of the application of the rubber dam. They complain of the buzzing of the engine as much as the actual pain. I believe if we will study these people, and develop a sympathy for them, getting their point of view, that we will eliminate a great deal of this dread of pain without the administration of so many drugs.

Dr. DeFord closing the discussion: It is entirely unnecessary to employ general anesthetics for every patient for whom we operate. But in all those cases in which the pain inflicted is severe enough to prevent ideal cavity preparation, or the removal of every trace of dental caries, of displacement of salivary concretions, and even when by means of comfortable operating considerable time can be saved to both the patient and operator, I believe in analgesia induction. Some dentists are in the habit of condemning everything advanced by other members of the profession that they themselves cannot do skilfully. Men timid about the administration of general anesthetics, and others who have not the temerity to try, find it more convenient to knock, oppose and condemn a procedure they have no practical knowledge of whatsoever. There are many conditions we meet with in daily practice that can be handled better by the employment of general anesthetics than in any other manner. For instance, a patient presents with a case of incipient acute septic apical pericementitis in a lower molar. This tooth is so sore that even the touch of the tongue causes excruciating pain. In a few hours an acute alveolar abscess will result, with its attendant and resultant concomitants. It is almost out of the question to enter the pulp chamber of such a tooth for vent of gases. Consequently the gum is painted with iodin, and the patient sent home to combat, the best he can, the severest pain which can be inflicted upon a mortal, as acute alveolar abscess will rapidly develop. By the use of somnoform or nitrous oxid, in two minutes the pulp chamber of such a tooth could be entered painlessly, drainage established, relief instantly afforded, three or four days of torture prevented, a chronic alveolar condition avoided.

During the winter a patient came to our clinic a distance of forty miles. We found deep saucer shaped cavities in five of the lower anterior teeth, gingival cavities, labially situated. They were so deep that the pulps would have died under any kind of a filling. They extended below the gum margins making it impossible to adjust the rubber dam. They were that sensitive that drying them with cotton caused the severest of pain. They could not be properly cut to retain arsenic with cement. What can be done in a case of this kind, especially when the patient has to come long distances? I administered somnoform just as for tooth extraction, and with a large bur opened into the pulp chamber of each one of these five teeth, and the time consumed from first inhalation was less than two minutes without the slightest pain or knowledge of the operation. Plenty of time remained to have removed all of these pulps with a broach, but the case was turned over to a student in order that he might continue the operation with pressure anesthesia for the experience.

I am sure you will be delighted with analgesia if you will try it. You can do more work and better work in the same ength of time. Some one has remarked that it is impossible for him to get patients to take a general anesthetic. These same patients would never have a rubber dam adjusted if they could help it. My method has been when I feel that analgesia is indicated, induce it, just as I would use any other instrument or appliances that seemed appropriate for the operation at hand. We do not stop and ask the patient if we should make an application of arsenic or cocain or iodin, or if we should adjust the rubber dam. We should be big enough and broad enough and courageous enough to do what is best for a given case, because we are in every sense of the word, surgeons, and just as other surgeons employ general anesthetics when they are indicated, so should we.

Dr. Reeves closing the discussion:

I have always been a firm believer in putting the patient in the right mental attitude for having work done. I have always been successful in preparing cavities, and I can truthfully say that during a period of a little over eight years, that I have used compressed air in this way. I have been very conservative in my statements as to results. Over seventy-five percent. of all cases under all conditions have been prepared without pain. The great advantage is its extreme simplicity and quickness. Before you have your instruments ready, the patient is prepared by your assistant, and you can go right to work. I work as fast as the engine runs, and I will prepare a cervical cavity complete inside of five minutes, while other cavities are finished in the same proportion. It is seldom that molar cavities take more than twenty minutes. When you can have the patient comfortable, I think it is worth while for you to make the effort.

